# Impact of obesity on outcomes in patients with sepsis

**DOI:** 10.1186/cc13236

**Published:** 2014-03-17

**Authors:** PP Dobesh, TR McGuire, DG Klepser, AL Himmelberg, DA Roberts, KM Olsen

**Affiliations:** 1 University of Nebraska Medical Center College of Pharmacy, Omaha, NE, USA

## Introduction

The purpose of this study was to evaluate the impact of obesity on outcomes in patients with severe sepsis. Since obesity is considered an inflammatory disease and is associated with elevations in several inflammatory mediators important in the outcome of sepsis, the relationship between obesity and outcome in septic patients was studied.

## Methods

This retrospective cohort study included all patients over the age of 40 with a confirmed diagnosis of severe sepsis and an ICU stay at our academic medical center from 1 January 2005 to 31 March 2011. Obesity was defined as a body mass index of 30 or greater. Data on other patient demographics and APACHE II score at the time of sepsis were collected from patient charts. Outcomes measured included inhospital mortality, development of acute respiratory distress syndrome (ARDS), days on mechanical ventilation, hospital cost, and length of stay.

## Results

We identified 824 patients who met the inclusion criteria for this study. Of these patients, 257 (31.2%) were classified as obese. The mean APACHE II score was similar between obese and nonobese patients (23.3 vs. 22.4; *P *= 0.068). Obese patients had a similar rate of in-hospital mortality (31.9% vs. 33.7%; *P *= 0.810) compared with nonobese patients, but a significantly higher rate of development of ARDS (49.4% vs. 34.4%; *P *< 0.001). Obese patients also had significantly more days on mechanical ventilation (6.2 days vs. 5.0 days; *P *= 0.005). There was no relationship between mortality in obese patients on mechanical ventilation (34.4% vs. 39.5%; *P *= 0.26) or ARDS (33.9% vs. 42.6%; *P *= 0.13) compared with nonobese patients. Hospital costs and length of stay did not differ between the groups.

## Conclusion

Obesity significantly increased the incidence of ARDS and days on mechanical ventilation in patients with sepsis. Previous work has reported that obesity is associated with elevations in inflammatory cytokines and adipokines, particularly IL-6, which is a known risk factor for ARDS. The higher rate of ARDS in obese patients with sepsis identifies a high-risk group where new therapies may be most beneficial and where new methods of preventing ARDS can be targeted.

